# Protective effect of rubber seed oil on human endothelial cells

**DOI:** 10.1007/s10735-024-10198-1

**Published:** 2024-06-19

**Authors:** Yujie Zhang, Fuchuan Huang, Yiran Wu, Linmei Jiao, Yun Wang, Tao Ding

**Affiliations:** 1 The First Affiliated Hospital of Kunming Medical University, No. 295 Xichang Road, Wuhua District, Kunming, Yunnan China; 2 Xishuangbanna Huakun Biotechnology Co., Ltd, Xishuangbanna Dai Autonomous Prefecture, Jinghong, Yunnan China

**Keywords:** Rubber Seed Oil, Atherosclerosis, Endothelial cells, Oxidized Low-Density Lipoprotein, Inflammation

## Abstract

**Objective:**

This study was conducted to characterize the antioxidant and anti-inflammatory properties of Rubber Seed Oil (RSO) against atherosclerosis (AS) through the study of the protective effects and mechanisms on human umbilical vein endothelial cells (HUVECs) injury induced by oxidized low-density lipoprotein (ox-LDL).

**Methods:**

HUVECs were treated with RSO, ox-LDL, RSO + ox-LDL, respectively, followed by cell activity testing, levels of IL-1β, IL-6, IL-10, TNF-α, ROS, NO, the mRNA expression of eNOS and protein expression of MCP-1, VCAM-1, eNOS, TLR4, NF-κB p65、p-NF-κB p65.

**Results:**

Compared with the ox-LDL group, cell viability, NO level and the expression of eNOS mRNA significantly increased. and the levels of pro-inflammatory factors such as IL-1β, IL-6, TNF-α, IL-10, ROS were significantly decreased, which was accompanied by decreases in TLR4 mRNA, TLR4, MCP-1, VCAM-1 protein expression, as well as the ratio of NF-κB p-p65/p65 in the group treated with 250 μg/ml ox-LDL + 50 μg/ml RSO, 250 μg/ml ox-LDL + 100 μg/ml RSO, 250 μg/ml ox-LDL + 150 μg/ml RSO.

**Conclusions:**

RSO can reduce the expression of pro-inflammatory mediators, oxidative factors involved in injured vascular endothelial cells, exhibiting anti-inflammatory and antioxidant properties HUVECs exposed to ox-LDL. In addition, it may alleviate endothelial cell damage by inhibiting the TLR4/NF-κB signaling pathway.

## Introduction

As the leading cause of mortality in developed countries, atherosclerosis (AS) is a systemic multifactorial disease characterized by lipid deposition, inflammatory factors aggregation, and vascular smooth muscle cells (VSMCs) proliferation accompanied by fibrous matrix hyperplasia (Grootaert and Bennett 2021). The accumulation of oxidized low density lipoprotein (ox-LDL) is an important event in the pathogenesis of atherosclerosis (Kattoor et al. 2019). When endothelial cells are damaged or subjected to metabolic stress conditions, it will lead to an increase in reactive oxygen species (ROS) production (Chatzizisis et al. 2007). Correspondingly, increased level of ROS can result in the production of ox-LDL (Förstermann and Sessa 2012). ox-LDL causes the increase of leukocyte adhesion molecules in endothelial cells, activates the apoptosis pathway, and leads to endothelial dysfunction, plaque instability and thrombosis (Kattoor et al. 2019). Hence, it may lead to the occurrence of acute cardiovascular disease (Miano et al. 2021). In recent years, the roles of inflammation and oxidative stress in atherosclerosis had attracted increased attention (Zhu et al. 2018; Poznyak et al. 2021; Kattoor et al. 2017).

Rubber seeds are one of the main by-products of natural rubber plantations, which are very rich in oil, called rubber seed oil (RSO), with 40–50% oil content of the seed kernel (Azócar et al. 2010). In recent years, some preliminary studies have concluded that RSO has no toxicity (Gandhi et al. 1990) and possesses increased catalase activities in fish (Deng et al. 2015) and radical scavenging abilities as a cosmetic (Lourith et al. 2014). In several studies, RSO exhibited anti-inflammatory property because it can reduce the level of proinflammatory factors (TNF-α and INF-γ) in fish and inhibit the production of NO, IL-1β, IL-6 in lipopolysaccharide-induced RAW 267.4 macrophages (Pi et al. 2019; Liu et al. 2022). However, the roles of RSO in human diseases are not well understood.

In this study, we expose human umbilical vein endothelial cells (HUVECs) to ox-LDL to simulate the development of AS pathology to study the effects of RSO on inflammatory and oxidative factor levels. Further, relevant signaling transduction pathways are studies to elucidate the mechanism of anti-AS effects. Our aim is to provide a theoretical basis for the further application of RSO in the treatment of human diseases.

## Methods

### Cell culture

The HUVECs cells (BeNa Culture Collection, Suzhou, China) were cultured in specialized culture medium (Procell Life Technology Co., Ltd, Wuhan, China) in a humidified atmosphere at 37℃ containing 5% CO_2_ and were sub-cultured every two to three days to maintain an exponential growth phase during experiments.

### Preparation for RSO

The RSO used in this study was provided by Huakun Biotechnology Co., Ltd. In Xishuangbanna, Yunnan, China. The seed oil was made from Brazilian rubber tree seeds and processed through raw material cleaning, drying, shelling, pressing, and refining. RSO was sterilized by filtration though a 0.2 µm membrane (Jiete Biofiltration Co., Ltd, Guangzhou, China) and dissolved in acetone (Wansheng Chuandong Chemical Co., Ltd, Chongqing).

### Cell viability testing

In the cell viability testing, cells were inoculated into the 96-well plate (Beyotime Biotechnology, Shanghai, China with a density of 1 × 10^5^ cells/well and cultured for 3 h until the cells adhered to the plate Then, the cells were treated with RSO in different concentration (25, 50, 75, 100, 125, 150, 200 µg/mL), as the previous study described (Liu et al. 2022), and ox-LDL (Solaybao Technology Co., Ltd, Beijing, China) at 12.5, 25, 50, 100, 150, 200, 250, 300 µg/mL, respectively, for 24 h.

After 24 h treatment, the original culture medium from the 96 well plate was extracted and 10μL CCK-8 reagent (Proteintech, Chicago, USA) was added to each well. After 1 h incubation, Optical Density (OD) was measured at 450 nm in the culture plate using a plate reader (BioTek, Vermont, USA).

### Determination of inflammatory cytokines

Supernatant from each treatment group described above was collected and stored at -20℃ until analyses. Levels of IL-1β, IL-6, IL-10, GSH and TNF-α were determined by an ELISA kit (Huamei Bioengineering Co., Ltd, Wuhan China) at 450 nm in triplicates (Kaval et al. 2022; Chen et al. 2021). Concentrations of inflammatory cytokines were calculated by standard curves and expressed as microgram or nanogram per milliliter per manufacturer’s instructions.

### Detection of intracellular ROS levels

The levels of intracellular ROS were analyzed using a ROS Assay Kit (Fu et al. 2023), a fluorescence enzyme-linked immunosorbent assay (Solaybao Technology Co., Ltd, Beijing, China). The DCFH-DA was diluted to 10 µmol/L and added to each well so that it can cover all cells. Next, the cells were incubated at 37℃ for 20 min. After discarding the culture medium, serum-free culture medium was used to wash the cells three times with gentle shaking. ROS levels were detected and plate reader, with parameter settings of 488 nm excitation wavelength and 525 nm emission wavelength (BioTek, Vermont, USA).

### Determination of NO

NO levels were measured with a NO detection kit, an enzyme-linked immunosorbent assay (Beyotime Biotechnology Co., Ltd, Shanghai, China). 60 μl of cell supernatant was added to each well in a 96 well plate with 5 μl NADPH, FAD, and Nitrate Reductase reagents incubated at 37 º C for 30 min. Then, the cells were incubated with LDH Buffer and LDH at 37 ℃ for 30 min. Next, 50 µl Griess reagent I and 50 µl Griess reagent II were added to each well at room temperature (20–30 ℃) for 10 min, and the OD value at 540 nm was measured with a plate reader (BioTek, Vermont, USA).

### RNA extraction and PCR

Cells were washed with PBS for three times after treatment. Total RNA was extracted from cells with Trizol (Solaybao Technology Co., Ltd, Beijing, China) and chloroform (Shandian Pharmaceutical Co., Ltd, Yunnan, China). The total RNA was collected in the aqueous phase after centrifugation ands precipitated by isopropanol (Jingrui Technology Co., Ltd, Yunnan, China). The pellet was washed with 75% ethanol, and redissolved in RNAase-free water. The concentration and purity of the total RNA were determined using a spectrophotometer. Quantitive RT-PCR (Sangon Biotech, Shanghai, China) was used measure the expression levels of genes are below (Table 1) in triplicate assays. Based on the threshold cycle (Ct) values of the target genes and internal control gene, the relative expression level of target genes in each group was calculated by the 2^−ΔΔct^ method.

**Table 1:** The primers of genes

Gene	Primer (5’-3’)	Product length
eNOS	Forward: GAAGCGAGTGAAGGCGACAATC	164
	Reverse: CCACCAGCACCAGCGTCTC	
TLR4	Forward: TGGTGGAAGTTGAACGAATGGAATG	191
	Reverse: GCAGCCAGCAAGAAGCATCAG	
MCP-1	Forward: CAGCAGCAAGTGTCCCAAAGAAG	114
	Reverse: TGCTTGTCCAGGTGGTCCATG	
VCAM-1	Forward: GTGACTCCGTCTCATTGACTTGC	134
	Reverse: AGGATTCATTGTCAGCGTAGATGTG	
NF-kB P65	Forward: CGCATCCAGACCAACAACAACC	80
	Reverse: TGGAAGCAGAGCCGCACAG	

### Western blot

After the cells were washed twice with PBS, 200 µl of lysing buffer was added to each well, and placed it on ice for 30 min; 4 ℃, 12,000 rpm, centrifugation for 30 min; Then the protein concentration was detected using the BCA kit (Biyuntian Biotechnology Co., Ltd, Shanghai, China), followed by heating in a metal bath at 95 ℃ for 15 min and cooling at room temperature for denaturation.

SDS-PAGE separated the total protein, and the protein was transferred to the polyvinylidene fluoride (PVDF) membrane (Millipore, Massachusetts, USA). The PVDF membrane was sealed at room temperature for 1 h on a shaking table using a sealing solution prepared with TBST and skimmed milk powder (Wandashan Dairy Industry Co., Ltd, Hulin China). Then washed it with TBST three times, and immersed it in the prepared primary antibodies at 4 ℃ overnight. Then, washed it with TBST three times. Next, put the membrane into the secondary antibodies, which were incubating for 1 h. Finally, protein imprinting on the PVDF film can be displayed with a visualizer. Meanwhile, data was analyzed by Image J.

Antibodies: eNOS (1:2000, ABclonal, Wuhan, China), NF-κB p65 (1:1000, ABclonal, Wuhan, China), MCP-1 (1:2000, Proteintech, Chicago, USA), TLR4 (1:1000, Proteintech, Chicago, USA), VCAM-1 (1:2000, Hua'an Biotechnology Co., Ltd, Hangzhou, China), p-NF-κB p65 (1:500, Affinity Biosciences, Cincinnati, USA).

### Statistical analysis

All experimental data were statistically analyzed using GraphPad Prism. Comparison between groups were conducted using conventional one-way ANOVA and the t-test. The statistical results were expressed as mean ± standard deviation (χ ± s), and *P* < 0.05 was considered statistically significant.

## Results

### RSO dissolves in acetone

In order to improve the effectiveness of RSO on cells, several solvents were screened for tehri ability to dissolve RSO. The results showed that RSO was insoluble in both DMSO (Dimethyl sulfoxide) and alcohol, the more commonly used solvent in cellular studies but soluble in acetone as previously reported (Agency for Toxic Substances and Disease Registry (ATSDR) 2022).

### Effects of acetone on HUVECs

**Fig. 1:**
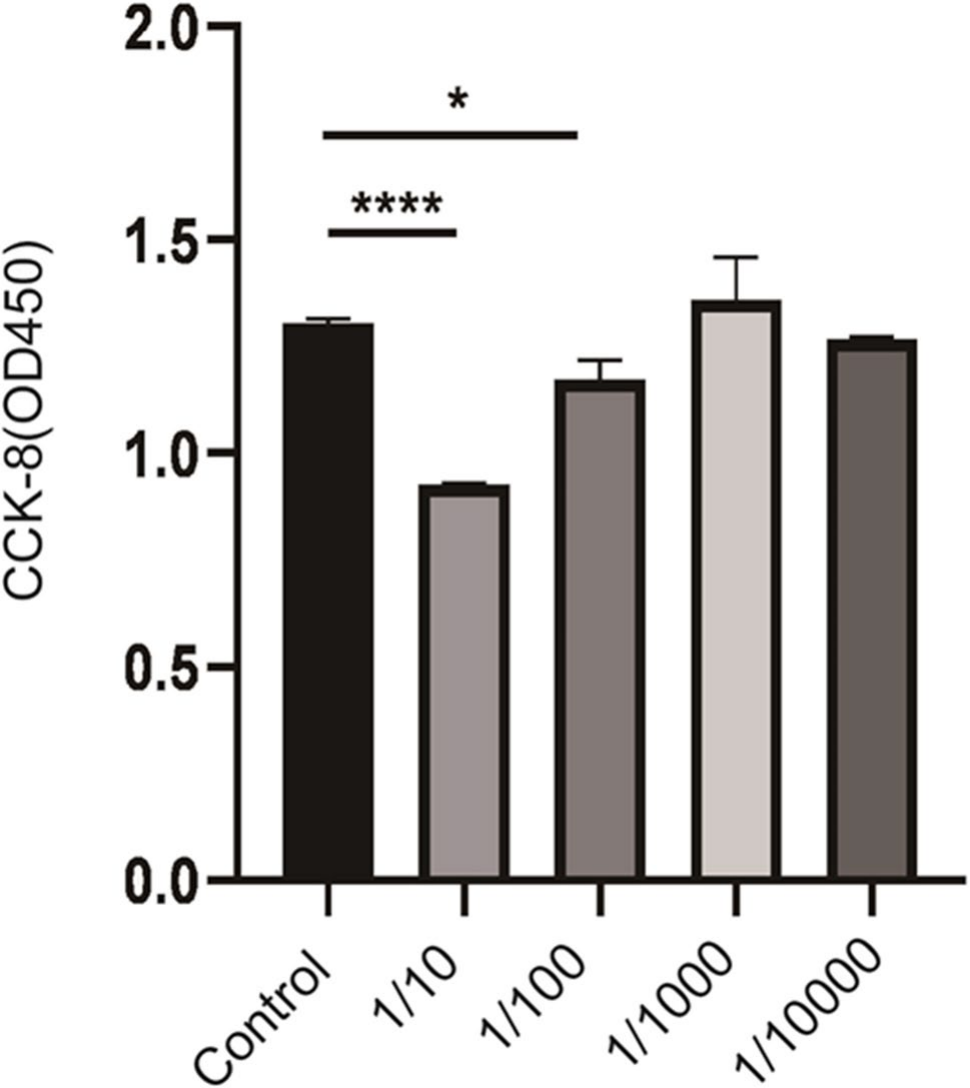
Effects of 0.01%, 0.1%, 1%, and 10% acetone on HUVECs viability after 24 h treatment

To determine effects of acetone as solvent on HUVECs, preliminary experiments were performed. HUVECs were treated with 0.01%, 0.1%, 1%, and 10% acetone (Wansheng Chuandong Chemical Co., Ltd, Linhai, China) and cell viability was assessed after 24 h treatment. It was found that acetone with concentration of less than 0.1% (v:v) had no significant effect on the cell viability of HUVECs (Fig. 1). Therefore, 0.1% acetone was selected to dissolve RSO in experiments reported here.

### The effects of RSO and ox-LDL on the viability of HUVECs

**Fig. 2:**
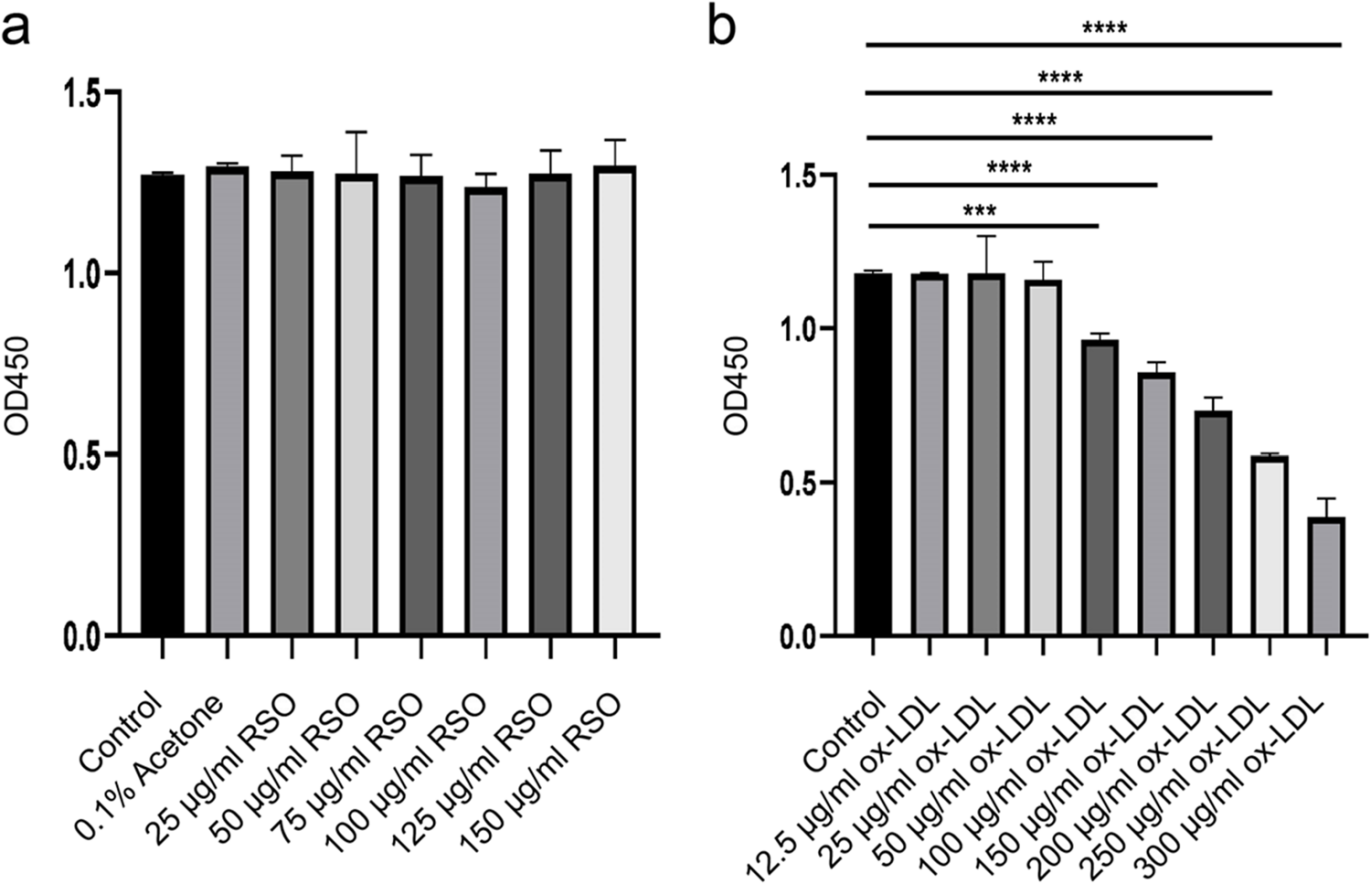
Effect of RSO, ox-LDL on the viability of HUVECs. (**a**) HUVECs cells were treated with RSO in different concentrations (0, 25, 50, 75, 100, 125, 150 μg/ml) for 24 h. (**b**) HUVECs cells were treated with ox-LDL in different concentrations (0, 12.5, 25, 50, 100, 150, 200, 250, 300 μg/ml) for 24 h. CCK-8 kit was used to detect the cell viability of different groups. (**** means *P* < 0.0001)

To investigate the effects of RSO on HUVECs and reduce errors caused by operations or measurements, 0, 25, 50, 75, 100, 125, 150 μg/ml of RSO in 0.1% acetone were used to treat these cells in triplicates and CCK-8 kit was used to assess cell viability. The results demonstrated that there was no significant difference in cell viability between these groups (*P* > 0.05), indicating that RSO has no cytotoxicity on HUVECs (Fig. 2a). However, we need to choose RSO concentrations that have little influence on HUVECs cell viability, but there should be a certain difference to avoid similar results in different groups for subsequent experiments. Therefore, we chose the RSO at a concentration of 50, 100, and 150 μg/ml.

HUVECs were co-cultivated with ox-LDL in different concentrations (0, 12.5, 25, 50, 100, 150, 200, 250, 300 μg/ml) for 24 h to determine the appropriate concentration of ox-LDL for simulating the damage process of endothelial cells during arteriosclerosis in the human body. And 100 μg/ml, 150 μg/ml, 200 μg/ml, 250 μg/ml, 300 μg/ml ox-LDL can significantly decrease the cell viability of HUVECs (*P* < 0.01) in a dose-dependent manner. The cell viability of the 250 μg/ml ox-LDL treatment group was close to the half of the control group which was selected for the subsequent experiments (Fig. 2).

### RSO protect HUVECs from ox-LDL induced cell death

**Fig. 3:**
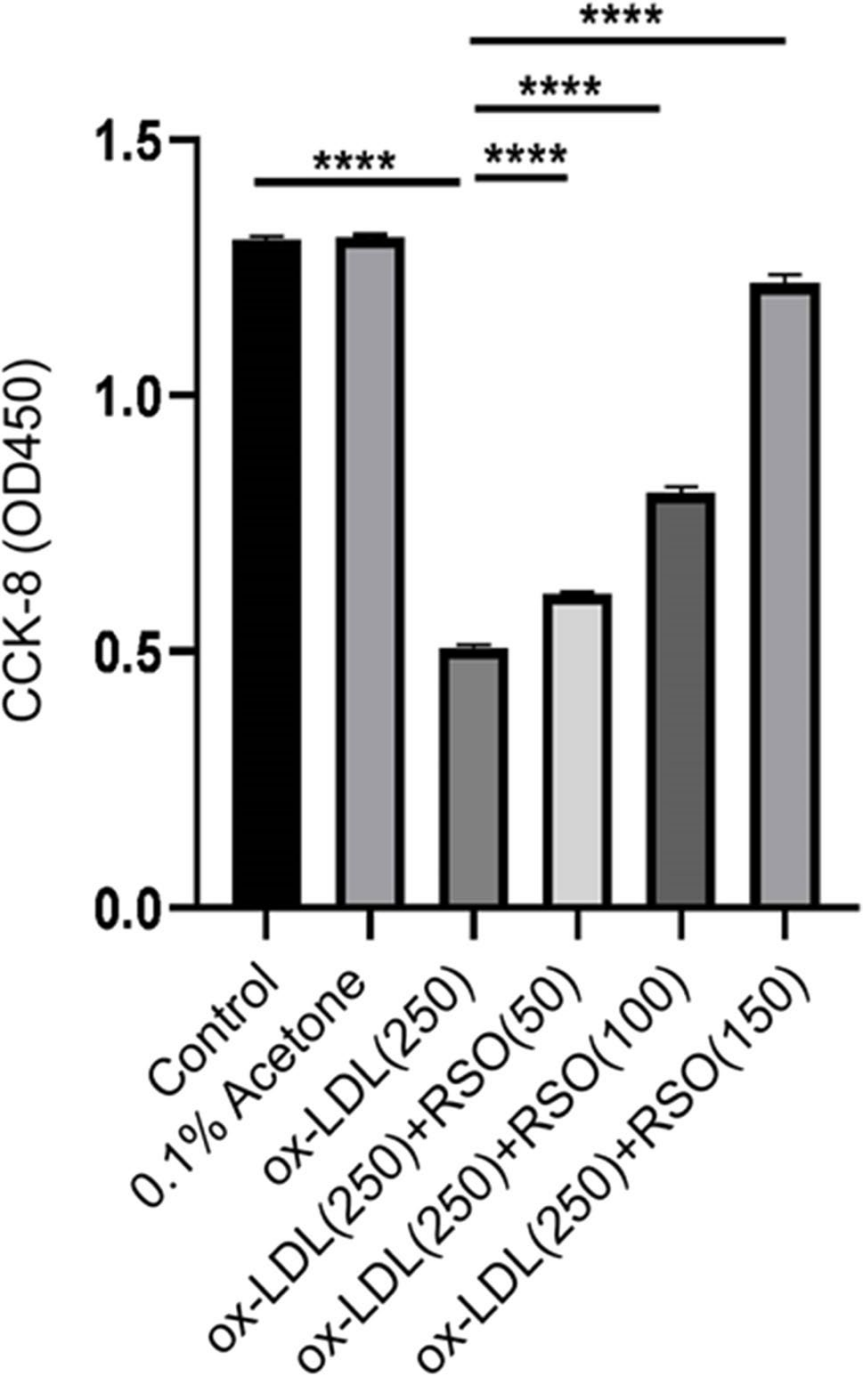
Effect of RSO on ox-LDL-induced cell death in HUVECs. Cells were treated with different intervention concentrations for 24 h. (**** means *P* < 0.0001)

RSO treatment demonstrated dose dependent protective effect compared against ox-LDL induced cell death (*P* < 0.01, Fig. 3).

### Effect of RSO on inflammatory factors in the ox-LDL treated HUVECs

**Fig. 4:**
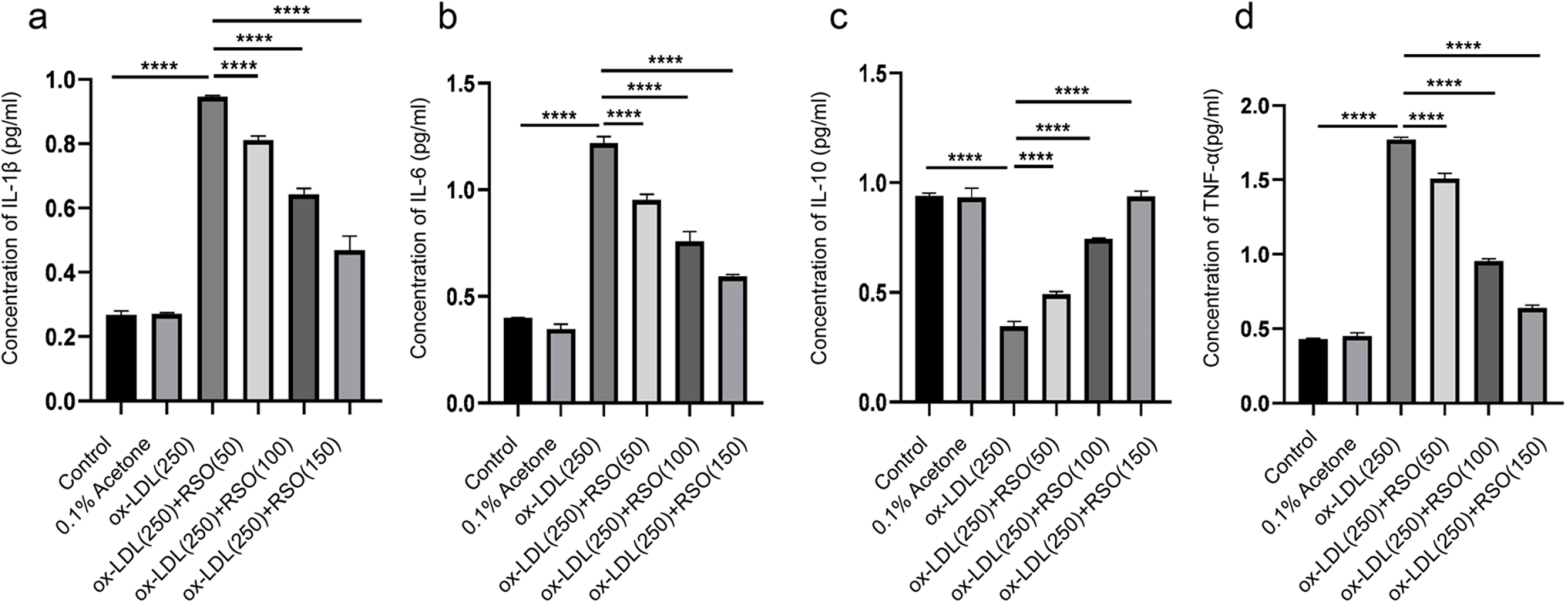
Effect of RSO on inflammatory factors of ox-LDL-induced HUVECs. ELISA kit was used to detect the levels of (**a**) IL-1β, (**b**) IL-6, (**c**) TNF-α and (**d**) IL-10 in the supernatant of each group of cells. (****means *P* < 0.0001)

Compared with the control group, the concentrations of inflammatory factors IL-1β、IL-6、TNF-α elevated significantly in the HUVECs treated with ox-LDL, while IL-10 decreased (*P* < 0.01) (Fig. 4) and treatment with RSO dose dependently reverse the changes of these inflammatory factors (*P* < 0.01) (Fig. 4).

### Effect of RSO on the expression of vascular adhesion factor of ox-LDL-treated HUVECs

**Fig. 5:**
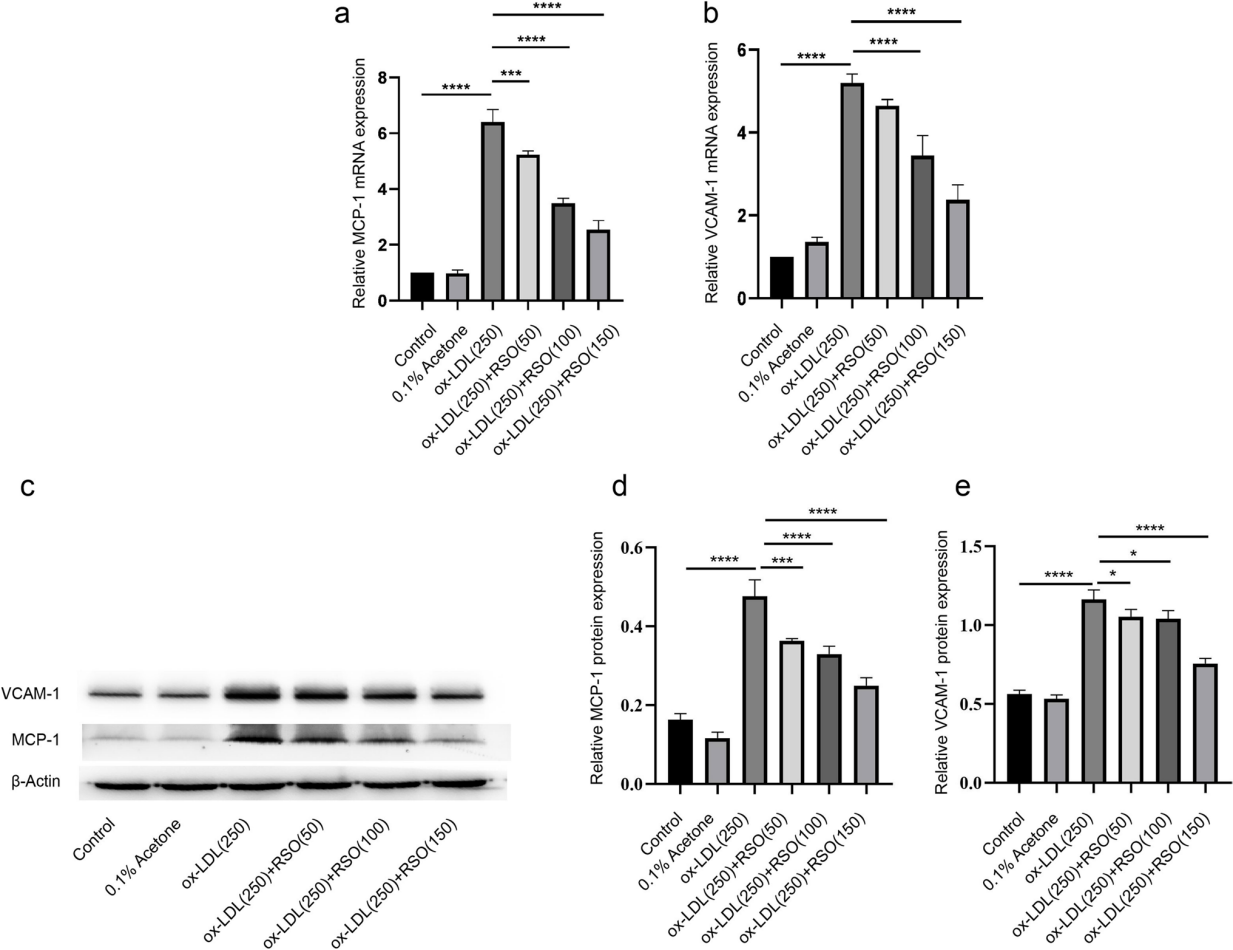
The effect of RSO on MCP-1 and VCAM-1 levels of ox-LDL-induced HUVECs. mRNA expression of **(a**) MCP-1 and (**b**) VCAM-1 in ox-LDL-induced injured HUVECs treated with and without RSO was detected by PCR, and the protein expression level as detected by western blot (**c**) and elative protein expression levels shown in (**d**) for MCP-1 and (**e**) for VCAM-1. (*means *P* < 0.05, ** means *P* < 0.01)

RSO treatment reduced the relative mRNA and protein expression levels of monocyte chemoattractant protein (MCP-1) and VCAM-1 in ox-LDL-treated cells (*P* < 0.001) (Fig. 5).

### The effect of RSO on ROS level of ox-LDL-treated HUVECs

**Fig. 6:**
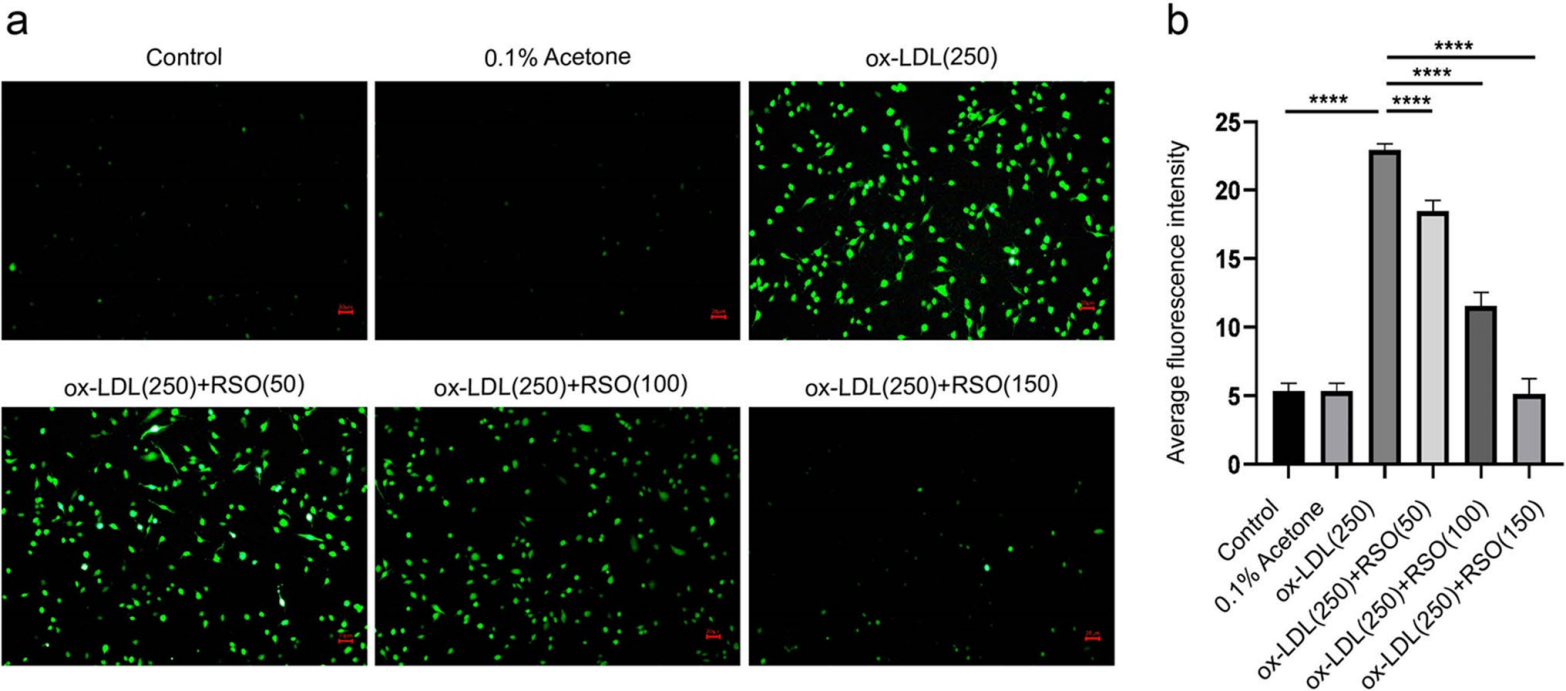
The effect of RSO on ROS levels in ox-LDL-induced HUVECs. (**a**) Intracellular ROS under fluorescence microscopy and (**b**) the data analyzed by Image J. (****means *P* < 0.0001)

The results showed that the ROS level in ox-LDL-induced injured cells was significantly higher than that of the control (*P* < 0.01) and RSO treatment reverse such increase in a dose dependent manner (50, 100, 150 μg/ml) (*P* < 0.01) (Fig. 6).

### The effect of RSO on NO content and eNOS levels in ox-LDL-treated HUVECs

**Fig. 7:**
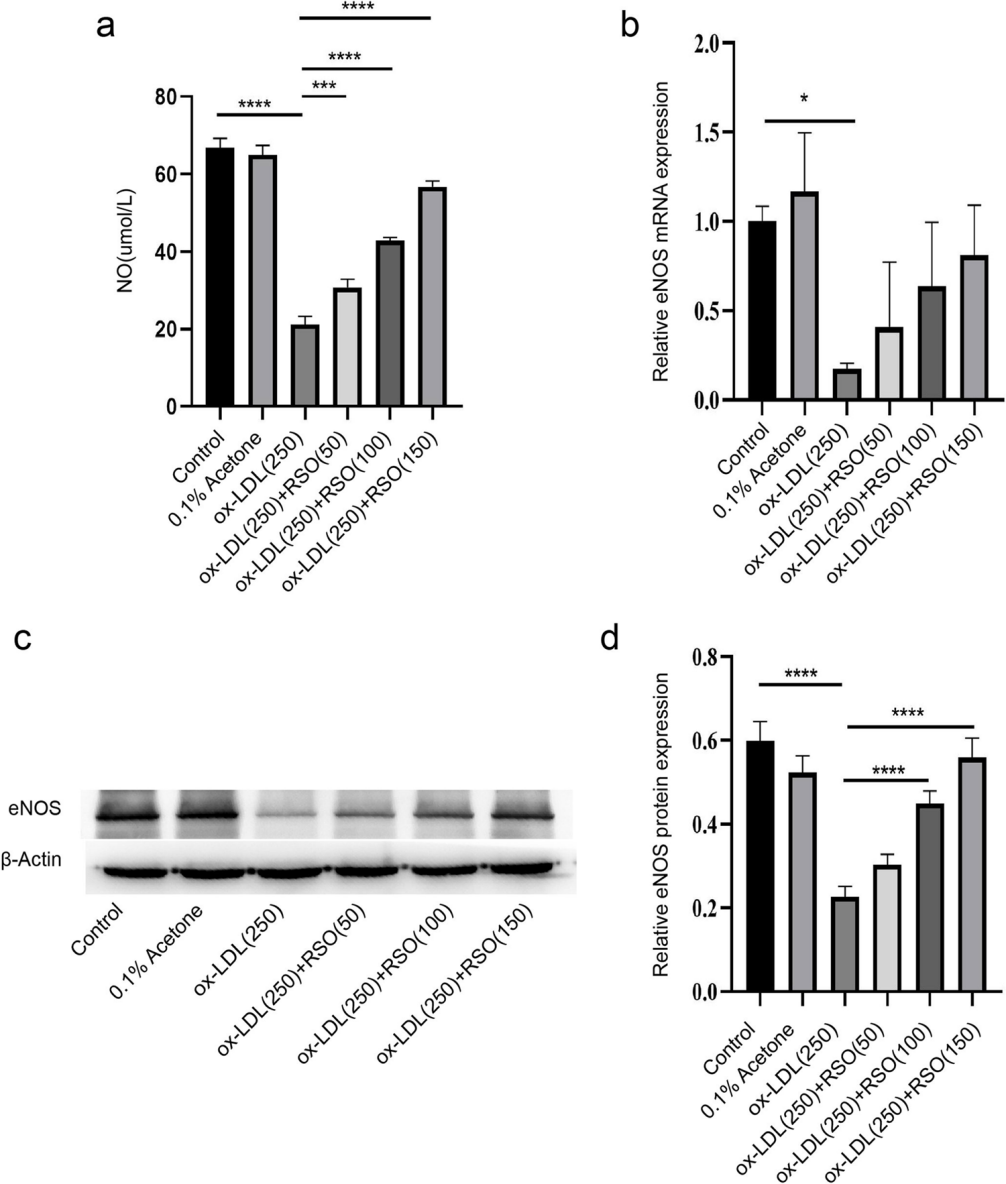
The effect of RSO on NO content and eNOS levels of ox-LDL-induced HUVECs. (**a**) NO levels (**b**) RNA expression of eNOS (**c**) Protein expression of eNOS and (**d**) the reltive protein expression levels. (* means *P* < 0.05, **** means *P* < 0.0001)

NO content, RNA and protein expression of eNOS in cells treated with ox-LDL were significantly lower than those in the control group (*P* < 0.01) (Fig. 7). RSO treatment could significant reverse the reduction of the protein levels induced by ox-LDL (*P* < 0.01) (Fig. 7a, b) but not significantly reverse at the mRNA levels (*P* > 0.05) (Fig. 7d).

### The effect of RSO on TLR4/NF-κB pathway in HUVECs

**Fig. 8:**
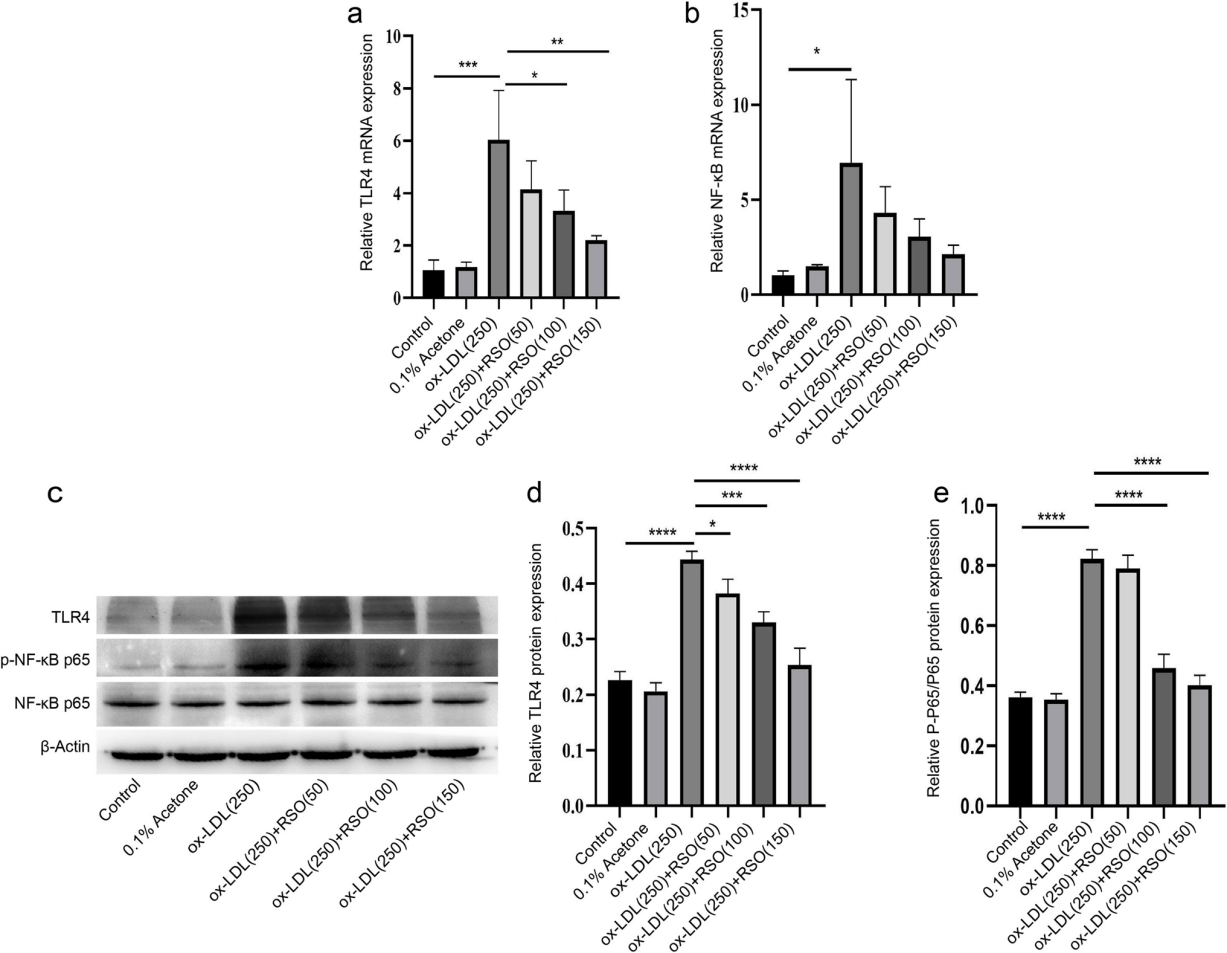
Effect of RSO on the TLR4/NF-κB pathway of ox-LDL-induced HUVECs. The RNA expression of (**a**) TLR4 and (**b**) NF-κB p65 detected by PCR. (**c**) The protein expression of TLR4 and NF-κB p65 measured by western blot, with β-Actin as the internal reference protein for normalization, and the relative expression of (d) TLR4 and (**e**) NF-κB p65 (* means *P* < 0.05, ** means *P* < 0.01, *** means *P* < 0.001, **** means *P* < 0.0001)

250 μg/ml ox-LDL treated cells demonstrated a significant increase in TLR4 and NF-κB p65 protein expression levels (*P* < 0.01), which was reversed dose dependently by RSO (*P* < 0.05). Further the protein expression levels of TLR4 and the ratio of NF-κB p-p65/p65 were increased in the ox-LDL treated group (*P* < 0.01) which were dose-dependently reversed by RSO (*P* < 0.05) (Fig. 8).

## Discussion

In the case of disturbed blood flow, endothelial cells and their tight junctions leak, which promotes the uptake of plasma LDL accumulated under the endothelium and which in turn evolves into ox-LDL (Ketelhuth and Hansson 2011). It can act on endothelial cells to stimulate the secretion of chemokines such as MCP-1, IL-6 and IL-1β and up regulate the expression of adhesion molecules such as VCAM-1 on the cell surface (Kattoor et al. 2019; Khosravi et al. 2019; Jun et al. 2022; Munjal and Khandia 2020; Ridker et al. 2011; Lin et al. 2014; Preiss and Sattar 2007). Subsequently, endothelial cell activation occurs from the oxidation reaction of these inflammatory mediators, leading to the expression of P-selectin (Woollard and Chin-Dusting 2007), E-selectin (Wenzel et al. 1994), attracts the aggregation and adhesion of monocytes, and thus promote the progress of AS process. In this work, the anti-inflammatory effects of RSO were assessed by studying the levels of related inflammatory factors compared with ox-LDL- HUVECs which simulates atherosclerosis as described (Gao et al. 2016). Our results indicated that cells treated with ox-LDL have a higher level of pro-inflammatory factors which can be reversed by RSO treatment.

In addition to inflammation, oxidative stress also plays an important role in the pathogenesis and progression of AS (Batty et al. 2022). The vascular wall can produce endogenous ROS (Zhang et al. 2022). However, excessive ROS can cause oxidative stress and damage blood vessels (Kattoor et al. 2017) and transform LDL into ox-LDL, stimulating the formation of foam cells and the release of matrix metalloproteinases (MMPs) which can promote the infiltration and migration of inflammatory mediators, and then destroy the normal structure of tissues (Yu et al. 2017; El Hadri et al. 2021). However, endothelial derived NO is a molecule that is catalyzed by eNOS and has various biological activities such as dilating blood vessels, reducing ROS, inhibiting platelet and leukocyte adhesion, and it can play an important role in cardiovascular protection and possess anti-AS functions (Wang et al. 2022). However, if the eNOS expression of endothelial cells is abnormal, the generation of NO may be affected, leading to ROS disorders and loss of protection of vascular endothelial cells and ultimately leading to AS lesions (Hong et al. 2019). The ROS content of HUVECs significantly increased while NO and eNOS levels significantly decreased under the treatment of ox-LDL, which both of which RSO reversed in a dose dependent manner indicating that RSO can alleviate the oxidative damage of HUVECs induced by ox-LDL through its antioxidant properties.

TLRs signaling pathways are considered bridges between the immune system and inflammatory diseases such as AS (Koushki et al. 2021). As an important member of the TLRs family, TLR4 which can be activated by inflammatory factors (Rocha et al. 2016), increase in carotid and coronary AS plaques in mice and humans (Xu et al. 2001). Meanwhile, Monaco et al. (Monaco et al. 2004) found that activated NF-κB can be detected in injured endothelial cells during the development of AS plaques. NF-κB is the ultimate effector molecule of the TLR4 signaling pathway and is also overexpressed in many inflammatory related diseases (Yao et al. 2019). Activated NF-κB can regulate the expression of pro-inflammatory factors, chemokines, adhesion molecules, growth factors, etc., playing a core role in inflammation (Tedgui and Mallat 2006; Tang et al. 2015). Our results showed that upregulated TLR4 and NF-κB were observed in HUVECs treated with ox-LDL and were dose-dependent reverse by RSO. Base on our results, it is speculated that RSO may reduce the secretion of inflammatory factors, chemokines, and adhesion molecules caused by ox-LDL in HUVECs by inhibiting the TLR4/NF-κB signaling pathway, thereby exerting a protective effect on HUVECs.

However, our study has the following limitations: Firstly, compared to the complex pathological and physiological processes of AS and the bioavailability of RSO in the human body, there may be an apparent distinction between in vitro and in vivo. Secondly, besides the TLR4/NF-κB signal pathway, the other mechanisms involved still need to be elucidated.

## Conclusions

In summary, this work demonstrated that the anti-inflammatory and antioxidant effects of RSO on the AS cellular model were associated with the secretion of NO, IL-10 and attenuation of TNF-α, IL-1β, IL-6, MCP-1 and VCAM-1 induced by ox-LDL treatments which could be due in part to the the reduction of the induced expression of TLR4/NF-κB. Our studies identified RSO as a potential AS protective drug and our results may provide new insight into preventive targets for people at isk of suffering from AS.
